# Morphological Effects of HA on the Cell Compatibility of Electrospun HA/PLGA Composite Nanofiber Scaffolds

**DOI:** 10.1155/2014/308306

**Published:** 2014-02-26

**Authors:** Adnan Haider, Kailash Chandra Gupta, Inn-Kyu Kang

**Affiliations:** Department of Polymer Science and Engineering, School of Applied Chemical Engineering, Kyungpook National University, Daegu 702-701, Republic of Korea

## Abstract

Tissue engineering is faced with an uphill challenge to design a platform with appropriate topography and suitable surface chemistry, which could encourage desired cellular activities and guide bone tissue regeneration. To develop such scaffolds, composite nanofiber scaffolds of nHA and sHA with PLGA were fabricated using electrospinning technique. nHA was synthesized using precipitation method, whereas sHA was purchased. The nHA and sHA were suspended in PLGA solution separately and electrospun at optimized electrospinning parameters. The composite nanofiber scaffolds were characterized by FE-SEM, EDX analysis, TEM, XRD analysis, FTIR, and X-ray photoelectron. The potential of the HA/PLGA composite nanofiber as bone scaffolds in terms of their bioactivity and biocompatibility was assessed by culturing the osteoblastic cells onto the composite nanofiber scaffolds. The results from *in vitro *studies revealed that the nHA/PLGA composite nanofiber scaffolds showed higher cellular adhesion, proliferation, and enhanced osteogenesis performance, along with increased Ca^+2^ ions release compared to the sHA/PLGA composite nanofiber scaffolds and pristine PLGA nanofiber scaffold. The results show that the structural dependent property of HA might affect its potential as bone scaffold and implantable materials in regenerative medicine and clinical tissue engineering.

## 1. Introduction

In the modern research world, electrospinning is one of the most frequently used techniques for the preparation of nonwoven fibrous materials with an ultrafine diameter (ranging from few nanometers to several hundred nanometers or even micrometers), high surface area per unit mass, and small interfibrous pore size [1–3]. Electrospun nanofiber mats of biocompatible polymers are of particular interest to bioengineers for potential applications in the fields of protein purification, drug delivery, enhanced immobilization, adhesion of biomacromolecules or cells, wound dressing, and so forth [4, 5]. Since pure polymers often display poor properties, they are generally loaded with certain nanomaterials to improve or add new properties. For instance, loading of silver or zinc oxide nanoparticles to polymer scaffolds has been reported to impart antibacterial and antifungal properties, along with better mechanical properties [6].

Over the last decade, the increased knowledge of the bone composition and hierarchical structure of the bone matrix has accelerated the research in bone tissue engineering [7]. New bioengineered and biomimetic systems, for example, nanocomposites of mineralized hydroxyapatite (HA) nanocrystals and collagenous fibers [8], were synthesized for the regeneration of bone. Numerous *in vivo *and *in vitro* studies have been carried out to examine the biocompatibility of HA nanocrystals with bones and teeth. The results of these studies have allowed researchers to use HA as a bone substituent [9, 10] with either natural or synthetic polymer. Therefore, it is one of the most reliable and frequently used implant materials in bone tissue engineering [11–13]. The biocomposites of HA/polymer scaffolds have shown improved bone cell responses both *in vitro* and *in vivo* because of their easy handling when polymer scaffolds are used as supports. Although the role of HA in the bone forming process is well established, the utilization of HA within the biopolymer matrix has remained limited [14, 15]. Regardless of the extensive literature on the synthesis of nanosize HA with a range of morphologies, such as spheres, nanorods, and nanofibers, very few reports are available on combining hydroxyapatite nanorods (nHA) with biocompatible polymers. Mostly, these reports elaborated the impact of nHA on the physical properties of the composites while leaving behind the important biological properties of the composite, such as their regenerative potential in terms of cell adhesion, proliferation, and so forth. Among the biocompatible polymers, poly(lactide-co-glycolide) (PLGA) is one of the most important materials used for blending with different types of biofillers owing to its unique property by which its time of biodegradation can be varied simply by varying the monomer ratio in its copolymer. The duration and degree of PLGA degradation is a very important factor for determining the usefulness of this material for particular applications [16, 17]. PLGA with 15 mol% glycolide is preferred for use as a bone implant material due to the fact that regeneration process is slow and takes a long time; the complete degradation of PLGA (85 : 15 molar ratio) takes five to six months [16, 17]. In addition to its use as an implant material, PLGA has also been used as a drug carrier owing to its increased inertness and tendency to restore soft tissue [18].

The objective of this study is to evaluate the effects of different HA morphological structures on the behavior of MC3T3-E1 (osteoblast) cells in terms of cell adhesion, spreading, and proliferation. Electrospun nHA/PLGA and sHA/PLGA composite nanofiber scaffolds and pristine PLGA nanofiber scaffold were prepared. The prepared nanofiber scaffolds were extensively characterized using a range of spectroscopic techniques before assessing their *in vitro* response to osteoblast (MC3T3) cells and their potential for future bone grafting and implanting materials.

## 2. Materials and Method

Poly(lactide-co-glycolide) (PLGA (glycolide 15 mol%) average molecular weight (Mw) 240000), silver nitrate (AgNO_3_), and spherical hydroxyapatite (sHA) were purchased from Sigma-Aldrich. Osteoblast (MC3T3-E1) cell line was obtained from Korea Cell Bank. The 5-bromo-2-deoxyuridine (BrdU) and alizarin red staining kits were supplied by Roche Molecular Biochemicals, USA, and Millipore, USA, respectively, whereas fetal bovine serum (FBS) and penicillin G-streptomycin were acquired from Gibco, Japan. All reagents and chemicals in this study were used as received.

### 2.1. Solution Preparation and Electrospinning

PLGA in the concentration range of 5 to 20 wt% was dissolved in a binary mixed solvent (THF and DMF in 3 : 1 ratio) and the solution was stirred at room temperature overnight until complete dissolution. The solution prepared was then subjected to electrospinning. For electrospinning, the PLGA solution was placed into a 10 mL glass syringe fitted with a needle with an internal diameter of 0.9 mm. A typical electrospinning assembly consists of four components: (i) a pump, which holds the syringe containing polymer solution (the pump allows controlled outflow of the polymer solution), (ii) a high voltage supply (1 to 50 Kv), (iii) a metallic capillary (needle) connected to a syringe and a positive voltage, and (iv) a metallic collector (flat or rotation drum) connected to a negative voltage. Electrospinning begins when a high electric current is generated from the power supply. As the solution moves to the tip of the needle, the hemispherical shape of the droplet is destabilized by charges that accumulate on its surface and is converted to Taylor's cone. At a critical voltage, the electric forces overcome the surface tension on the droplet and an ultrafine jet is produced from the tip of Taylor's cone and collected on a collector. The optimized electrospinning conditions used in this study were tip to collector distance 20 cm, applied voltage 20 kV, and flow rate 1 mL/h. The electrospun nanofiber scaffolds collected on the collector were removed from the collector and dried overnight at 40°C to remove the solvent. The same procedure was adapted for the preparation of electrospun sHA/PLGA and nHA/PLGA composite nanofiber scaffolds, in which 1 wt% sHA and nHA were added separately to the polymer solution prior to electrospinning. The solutions were stirred overnight using a magnetic stirrer for the complete dispersion of sHA and nHA in the polymer solution.

### 2.2. Synthesis of Hydroxyapatite (HA) Nanorods

nHA were synthesized via chemical precipitation process [19]. Briefly, 400 mL (NH_4_)_2_PO_3_ and 300 mL CaNO_3_·4H_2_O solutions were prepared separately by dissolving 19.75 g of (NH_4_)_2_PO_3_ and 57.5 g of (CaNO_3_)·4H_2_O into distilled water. The pH of the (CaNO_3_)·4H_2_O solution was adjusted to 10.4 with NH_4_OH. After pH adjustment, both solutions were mixed dropwise with each other with continuous stirring. During the mixing of the two solutions white precipitates were formed. The precipitates formed were aged for four days to form nHA. The synthesized nHAs were washed with distilled water until the pH reached 7. The water surrounding the HA was replaced with 1-butanol to prevent nHA from aggregation during the drying process. The precipitates were then dried at 80°C and calcined at 500°C for 4 h to remove the rudimental organic compounds [19].

### 2.3. Characterization

The viscosity of the PLGA polymer solutions in the binary solvent (THF : DMF 3 : 1 ratio) was measured using a viscometer (Brookfield viscometer DV-II pro) at room temperature with spindle number 6 at 100 revolutions per minute (rpm). The morphology of the electrospun nHA/PLGA, sHA/PLGA, and PLGA nanofiber scaffolds and the cell adhered scaffolds was evaluated by field emission scanning electron microscopy (FE-SEM, 400 Hitachi, Tokyo, Japan). X-ray diffraction (XRD, Rigaku D-MAX IIB, Tokyo, Japan) of the nHA and sHA was carried out between 20 and 80° 2*θ* at 40 kV and 30 mA. The Fourier transform infrared (FTIR, Mattson, Galaxy 7020A) spectra of nHA, nHA/PLGA, and sHA/PLGA composites and PLGA nanofiber scaffolds were recorded. Prior to analysis, the samples were mixed with KBr and shaped into pellets under a hydraulic pressure. The presence of nHA and sHA nanoparticles in the electrospun nHA/PLGA, sHA/PLGA composite nanofiber scaffolds was studied by transmission electron microscopy (TEM, H-7600, Hitachi, Japan). The nHA/PLGA and sHA/PLGA composite nanofiber scaffolds were collected during the electrospinning process onto carbon grids, which were fixed to the collector. The nHA, sHA samples for TEM measurement were prepared by suspending the nHA and sHA into DI water and collecting these onto a carbon grid. The samples were dried at room temperature before analysis. The qualitative and quantitative chemical analyses of the nHA/PLGA, sHA/PLGA, and PLGA nanofiber scaffolds along with pristine nHA were carried out by X-ray photoelectron spectroscopy (ESCA, ESCA LAB VIG microtech Mt 500/1, Etc EAST Grinstead, UK), equipped with Mg K*α* radiation at 1,253.6 eV and a 150 W power mode at the anode. A survey scan spectrum was taken and the surface elemental compositions relative to the carbon were calculated from the peak heights taking into account the atomic sensitivity. The presence of HA in the HA/PLGA composite nanofiber scaffolds was carried out by energy dispersive X-ray spectroscopy (EDS/EDX). The data obtained was recorded in the form of peaks on the screen of the PC attached to the instrument.

### 2.4. Bioactivity and Cellular Response

#### 2.4.1. Cell Culture

To examine the interactions of the nanofibrous scaffolds with cells, circular nanofibrous scaffolds were fitted in a 24-well culture dish and subsequently immersed in a MEM medium containing 10% FBS and 1% penicillin G-streptomycin. One milliliter of a MC3T3 cell solution (5 × 10^5^ cells/cm^2^) was added to the sample sheet and incubated in a humidified atmosphere (5% CO_2_ and at 37°C) for 1 day to determine the cell adhesion on the nanofiber scaffolds. After incubation, the supernatant was removed, washed twice with PBS, and fixed with an aqueous 2.5% glutardialdehyde solution for 20 min. The sample sheet was then dehydrated, dried in a critical point drier, and stored for characterization.

#### 2.4.2. Cell Proliferation

The proliferation of MC3T3 osteoblast cells seeded on the nanofibrous scaffolds was determined using a colorimetric immune assay based on the measurement of BrdU, which was incorporated during DNA synthesis. The BrdU assay was performed according to the manufacturer's instruction. Briefly, after culturing the cells for 48 h, a BrdU-labeling solution was added to each well and was allowed to incorporate into the cells in a CO_2_ incubator at 37°C for a further 20 h. Subsequently, the supernatant in each well was removed by pipetting and washed twice with PBS. The cells were treated with 0.25% trypsin-EDTA and harvested by centrifugation of the cell solution at 1,000 rpm for 15 min. The harvested cells were mixed with a FixDenat solution to fix the cells and denature the DNA and incubated for 30 min. Subsequently, the diluted anti-BrdU-peroxidase (dilution ratio = 1 : 100) was added to cell and kept at 20°C for 120 min. After removing the unbound antibody conjugate, 100 mL of the substrate solution was added and allowed to stand for 20 min. The reaction was quenched by adding 25 mL of a 1 M H_2_SO_4_ solution. The solution was transferred to a 96-well plate and measured within 5 min at 450 nm with a reference wavelength of 690 nm using an ELISA plate reader (EL 9800). The blank corresponded to 100 mL of culture medium with or without BrdU.

#### 2.4.3. Alizarin Red Staining

Alizarin red staining of the MC3T3 osteoblastic cells was performed to examine the mineralization and differentiation. Briefly, after culturing the MC3T3 osteoblasts, the medium was aspirated without disturbing the cells. The culture dish with the osteoblastic cells was washed twice with PBS. The cells were then fixed with 10% formaldehyde and incubated for 15 min at room temperature. The fixative reagent was removed carefully and the cells were rinsed three times (10 minutes each) with distilled water to avoid disturbing the monolayer. After washing, the excess amount of water was removed and an alizarin red staining solution (1 mL/well) was added to the cells, and the samples were incubated for 30 min. When the staining time was complete, the excess amount of dye was removed from the stained cell by washing the samples four times with distilled water (5 min each) and gentle rocking. The digital images of the stained cell were acquired with a camera (Nikon E 4500, Japan).

#### 2.4.4. Von Kossa Assay

The calcium deposition of MC3T3-E1 cells was examined by Von Kossa staining. The cells were cultured for 15 and 21 days on nHA/PLGA and sHA/PLGA nanofiber scaffolds under the same conditions as those described in the alizarin red staining experiment. After incubation, the cells were washed three times with PBS for 5 min, fixed with 10% formaldehyde for 30 min, and washed three times with distilled water for 10 min. The fixed samples were treated with a 5% AgNO_3_ solution for 5 min under ultraviolet radiation. After removing the AgNO_3_ solution, the samples were washed with PBS twice followed by the addition of a 5% Na_2_S_2_O_3_ solution to the plate and allowing the plates to stand for 5 min. Finally the samples were washed twice with distilled water and the digital images of the stained cells were obtained.

### 2.5. Calcium Release

The Ca^+2^ ion release experiment was carried out to find out the difference between the ionization potentials of both nHA and sHA. The amount of Ca^+2^ ions released from each sample was determined by immersing the nHA/PLGA and sHA/PLGA (4 × 4 cm^2^, 0.2 g) nanofibrous scaffolds into 10 mL calcium-free PBS (pH 7.2) for different time periods. The amount of Ca^+2^ ions in the PBS solution was determined by inductively coupled plasma spectrophotometry (ICP, Thermo Jarrell Ash IRIS-AP).

### 2.6. Statistical Analysis

The results are displayed as the mean ± standard deviation. The statistical differences were determined using Student's two-tailed test. Scheffe's method was used for the multiple comparison tests at a level of 95%.

## 3. Results and Discussion

### 3.1. Preparation of the Nanofiber Scaffolds

**Figure 1:**
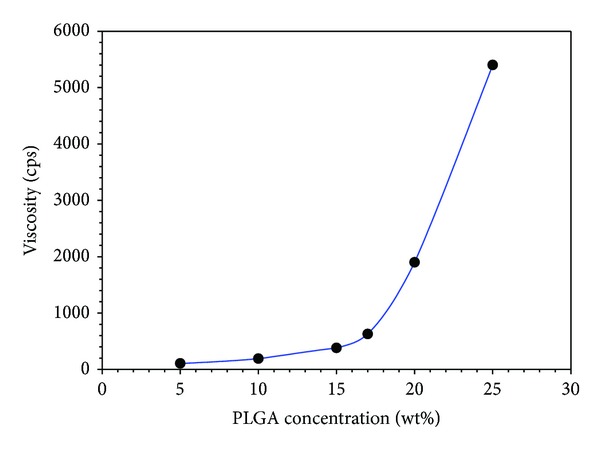
Relationship between the PLGA concentration in THF and DMF (3 : 1 ratio), and solution viscosity.

**Figure 2:**
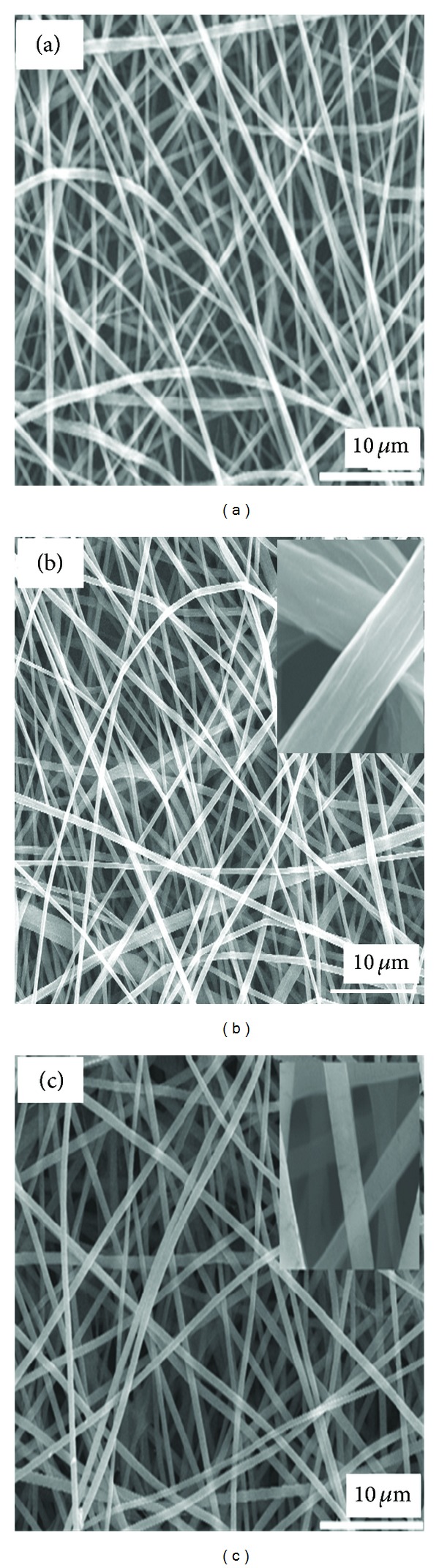
SEM micrographs of (a) 17 wt% PLGA nanofibers, (b) sHA/PLGA, and (c) nHA/PLGA composite nanofiber scaffolds.

Viscosity is one of the major factors for the preparation of smooth and bead-free fibers by electrospinning [20].  Figure 1 shows the increase in viscosity of the PLGA solution with increasing solution concentration (from 5 to 25 wt%). The 5 wt% solution did not show electrospun fiber and only droplets were obtained. The process under this condition is a characteristic of electrospraying rather than electrospinning. The formation of droplets was attributed to the insufficient molecular chain entanglements and low surface tension, which allowed the breakup of an electrically driven jet into droplets [21]. When the solution concentration was increased from 5 to 15 wt%, the morphology of the electrospun nanofibers changed from droplets to beaded nanofibers. The change in nanofibers morphology from droplet to beaded nanofibers with increasing concentration might be attributed to the increase in molecular chain entanglement, which prevented jet breaking [20]. On the other hand, the presence of decreased beads showed that the chain entanglements are still insufficient to make the jet completely stable. A further increase in the solution concentration (17 wt%) led to the formation of smooth nanofibers (Figure 2(a)), which is obvious from the fact that there was a higher level of polymer chain entanglement in the solution, which led to stable charged jet formation [21]. Stable jet formation is an indication that the viscosity (Figure 1) reached a critical (539.5 cps) value of 17 wt%, which avoided breakup (electrospraying occurs if there is breakup) of the polymer drop at the needle tip.

Figure 2 shows the SEM micrographs of the PLGA (a), sHA/PLGA (b), and nHA/PLGA (c) electrospun nanofiber scaffolds at optimized electrospinning parameters and 17 wt% PLGA solution. It is obvious from Figure 2(a) that, at 17 wt% of PLGA solution, uniform nanofibers were obtained. Using the optimized electrospinning parameters, 1 wt% sHA and nHA (with respect to the total PLGA) composite solutions were electrospun separately to make their respective electrospun composite nanofiber scaffolds. The smooth and beadless composite nanofibers of sHA (Figure 2(b)) and nHA (Figure 2(c)) showed that the addition of HA did not affect the polymer chain entanglement [20, 21].

### 3.2. TEM Study

**Figure 3:**
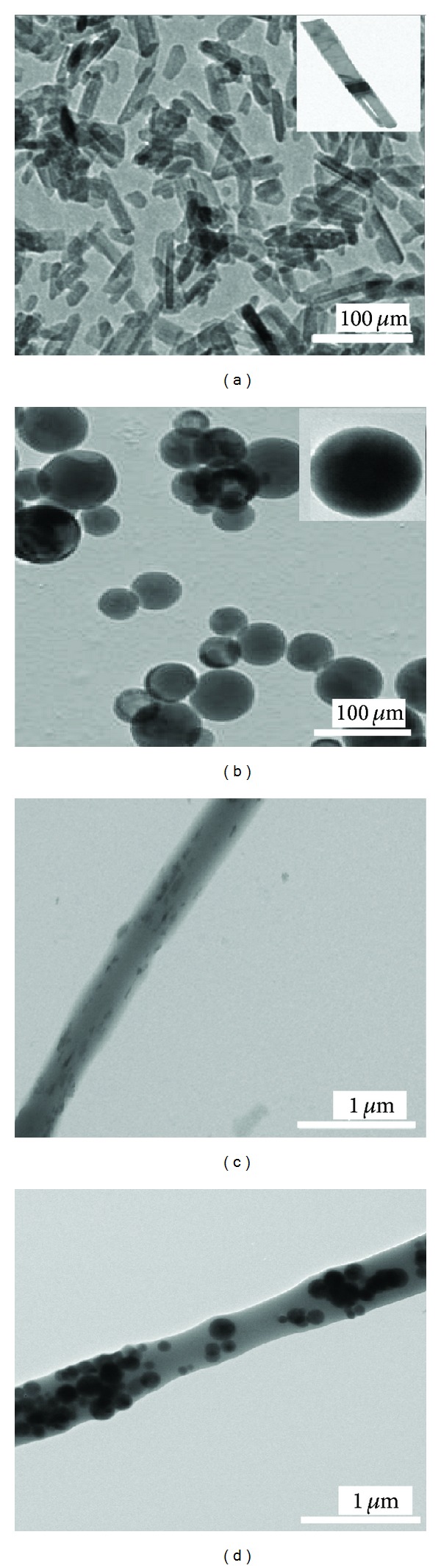
TEM images of the pristine particles (a) nHA and (b) sHA, and composite nanofiber scaffolds (c) nHA/PLGA and (d) sHA/PLGA.

TEM provides direct visualization of the filler materials in the polymer matrix. Figures 3(a)–3(d) show images of the nHA and sHA nanomaterials, as well as the nHA/PLGA and sHA/PLGA composite nanofiber scaffolds. The hydrophilic nHA (Figure 2(a)) and sHA (Figure 2(b)) were well dispersed in the PLGA matrix. In addition, as shown in Figure 3, hydrophilic nHA and sHA were fairly dispersed in the hydrophobic PLGA polymer matrix as a result of alternate vigorous stirring and sonication prior to electrospinning (Figures 3(c) and 3(d)).

**Figure 4:**
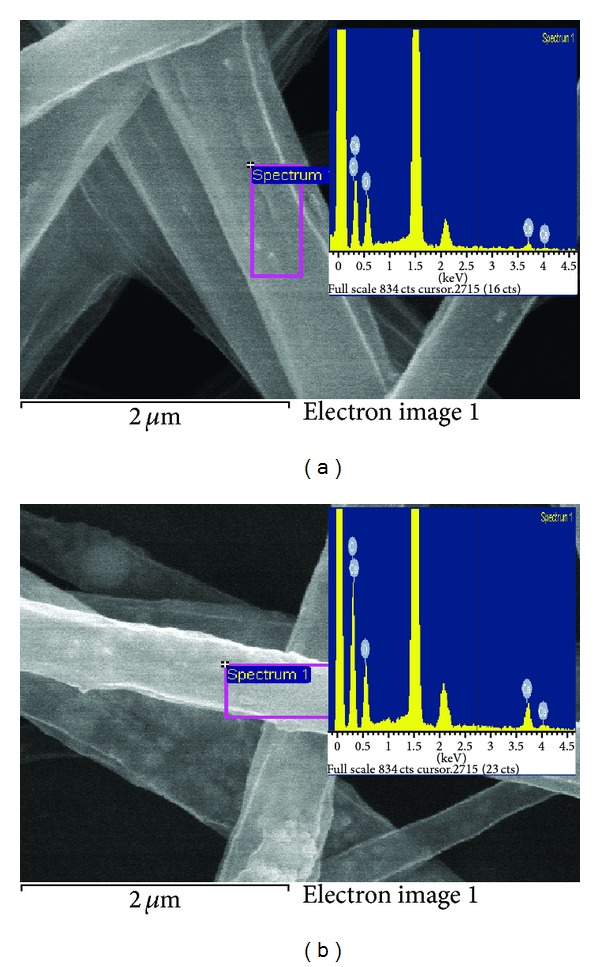
EDX scan spectra of (a) nHA/PLGA and (b) sHA/PLGA nanofiber scaffolds.

The EDX spectra of nHA/PLGA and sHA/PLGA nanofiber scaffolds (with sHA and nHA embedded in the polymer nanofiber) are illustrated in Figure 4. The presence of nHA and sHA in the nHA/PLGA and sHA/PLGA nanofiber scaffolds was confirmed by appearance of the characteristic peaks of calcium, phosphorous, and oxygen, which are the main components of hydroxyapatite. Additional carbon peaks were also observed in the EDX spectra of nHA/PLGA and sHA/PLGA nanofiber scaffolds. These carbon peaks were attributed to the presence of PLGA (Figures 4(a) and 4(b)). Hence, it is evident that nHA and sHA are present in both the nHA/PLGA and sHA/PLGA nanofiber scaffolds.

### 3.3. Spectral Analysis

**Figure 5:**
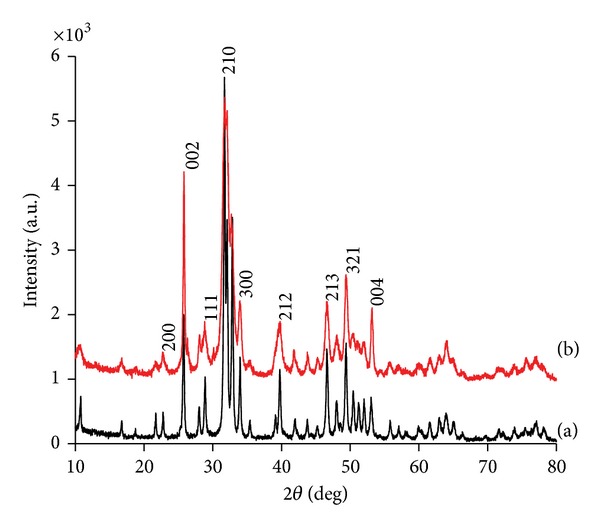
XRD profiles of the (a) sHA and (b) nHA.

Figure 5 illustrates the XRD profiles of sHA and synthesized nHA. The XRD profile of nHA shows the characteristic diffraction peaks at 26.1, 28.45, 30.1, 32.90, 35.97, 40.19, 41.82, 53.56, 55.75, 57.40, 69.12, 74.45, and 77.56°, corresponding to the 002, 102, 210, 112, 300, 212, 130, 213, 321, 004, and 104 planes, respectively, of the HA unit cell with hexagonal symmetry [22]. The unit cell parameters were determined to be *a* = 9.422 Å and *c* = 6.883 Å with the space group C63/m [22]. The peak positions in the sHA profile were observed at the same positions as in nHA. A distinguishable feature of the nHA XRD profiles is that the 002 peak is quite strong compared to sHA. This suggests that crystal stacking occurred along the 00l (*c*-axis) plane, which might be along the length of the nanorods. Similar results were reported for HA with a nanorod morphology [23]. The critical temperature used at which nHA alters its morphology from an amorphous phase to a crystalline phase was 500°C. The broadened nature of the nHA diffraction peaks compared to the sHA suggests that the grain size of nHA is in the nanometer scale and smaller than the sHA.

### 3.4. FTIR and XPS Studies

**Figure 6:**
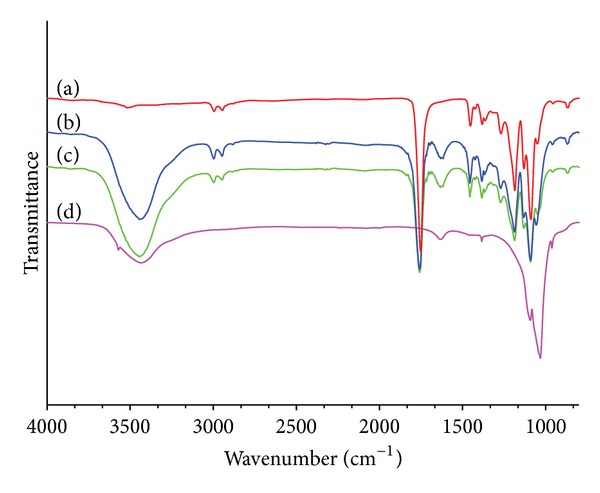
FTIR spectra of (a) PLGA, (b) nHA/PLGA, (c) sHA/PLGA nanofiber scaffolds, and (d) HA nanoparticles.

Figure 6 represents the FTIR spectra of (a) nHA, (b) nHA/PLGA, (c) sHA/PLGA, and (d) PLGA. A sharp peak at 1742 cm^−1^ that appeared in the PLGA polymer spectra is assigned to the C=O stretching of PLGA polymers. The peak at 1742 cm^−1^ also appeared in the nHA/PLGA and sHA/PLGA composite nanofiber scaffolds due to the presence of PLGA. The broadband at 3571 cm^−1^ in nHA was assigned to the stretching vibration of lattice hydroxyl (–OH) groups. The characteristic sharp bands of HA, which appeared in the regions of 1000–1100 cm^−1^, 562−570 cm^−1^, and 602 cm^−1^ in spectra of nHA, were assigned to the regular tetrahedral (PO_4_^−3^), P–O stretching, and O–P–O bending vibrations, respectively. These bands are present at their characteristic positions in the spectra of nHA/PLGA and sHA/PLGA nanofiber scaffolds [24]. The bands observed in nHA at 1384 cm^−1^ are due to the stretching vibration of carbonate [25, 26]. From the FTIR spectra it can be found out that nHA and sHA were successfully incorporated in the PLGA nanofiber scaffolds.

**Figure 7:**
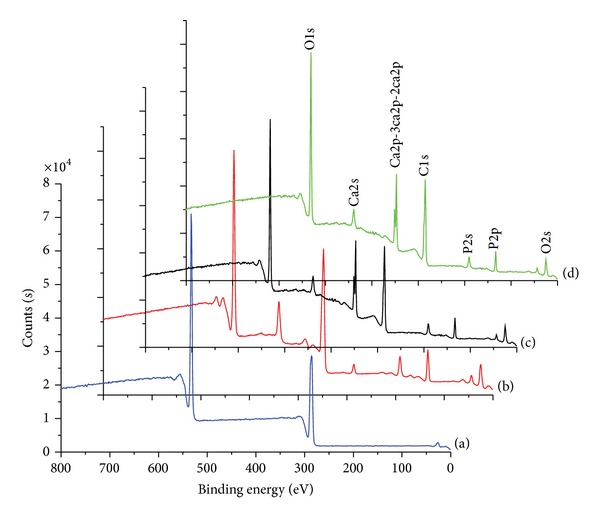
ESCA survey scan spectra of (a) PLGA, (b) nHA, (c) sHA/PLGA, and (d) nHA/PLGA nanofiber scaffolds.

The changes in the chemical composition and atomic weight% of pure HA in the nHA/PLGA and sHA/PLGA composite nanofiber scaffolds were investigated using ESCA.  Figure 7 shows the ESCA survey scan spectra of the pure nHA, nHA/PLGA, sHA/PLGA, and PLGA nanofiber scaffolds. The characteristic peaks of nHA appeared at 536.1 eV, 347.9, and 133.2 based on O1s, Ca2p, and P2p, respectively. The same corresponding peaks were observed with decreased intensities in the nHA/PLGA and sHA/PLGA nanofiber scaffolds.  Table 1 lists the chemical composition of the scaffolds calculated from the survey scan spectra. The calcium (17.8%) and phosphorous (12.6%) contents in pure nHA decreased to 3.0% and 3.2% in the nHA/PLGA and sHA/PLGA composite nanofiber scaffolds. Furthermore the amount of phosphorous was 4.9% and 4.7% in nHA/PLGA and sHA/PLGA nanofiber scaffolds, respectively. These results indicated that the nHAs are successfully embedded in the PLGA nanofiber matrix.

### 3.5. Bioactivity and Cellular Response

**Figure 8:**
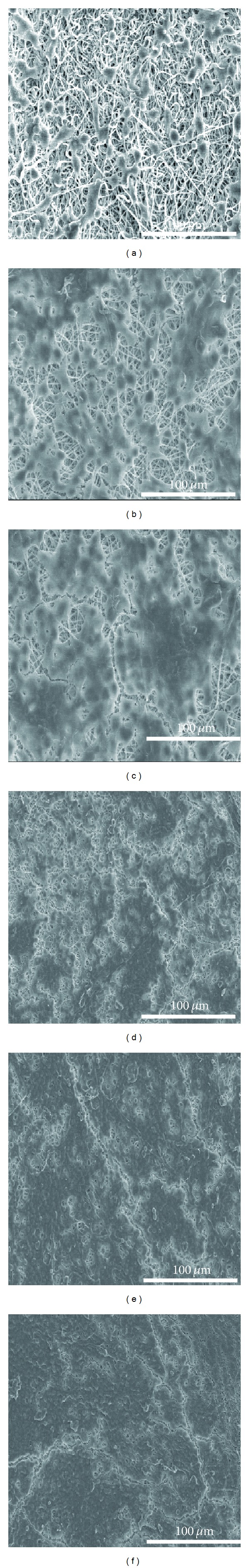
SEM micrographs of the cells adhered to the PLGA nanofiber scaffolds (a and d), sHA/PLGA (b and e), and nHA/PLGA (c and f) composite nanofiber scaffolds after 1- (a, b, and c) and 3- (d, e, and f) day incubation.

The clinical success of bone implants is dependent mainly on the formation of intimate contact between the implant surface and mineralized tissue. In order to achieve mineralization the adhesion, proliferation, and differentiation of osteoblasts must be optimized. The *in vitro *cell response to the nHA/PLGA, sHA/PLGA, and PLGA nanofiber scaffold was assessed in terms of cell adhesion.  Figure 8 depicts the FE-SEM images of the MC3T3 osteoblasts which adhered to the PLGA nanofiber scaffold (Figures 8(a) and 8(d)), sHA/PLGA (Figures 8(b) and 8(e)), and nHA/PLGA (Figures 8(c) and 8(f)) nanofiber scaffolds after 1- and 3-day culture. The SEM images revealed the bioactive properties of nHA with a preferential anchorage of osteoblast cells to the nHA/PLGA (Figures 8(c) and 8(f)) nanofiber scaffolds contrary to the sHA/PLGA (Figures 8(b) and 8(e)) and PLGA (Figures 8(a) and 8(d)) nanofiber scaffolds. Beside the quantification of the number of cells, cellular behavior is also a pivotal indicator to determine the potential applications of materials for tissue engineering applications. It was found, from the results depicted in Figure 8, that the adhesion of cells on the nHA/PLGA nanofiber scaffold was better compared to the sHA/PLGA and PLGA nanofiber scaffolds, suggesting that nHA is effective in accelerating interaction with osteoblastic cells better than its counterparts (sHA/PLGA and PLGA nanofibers scaffolds). The enhanced cell adhesion properties of nHA might be due to the fact that the fibrous nHA shows superior mechanical properties compared to its nonfibrous sHA counterparts [27]. The increased adhesion of osteoblastic cells to the scaffolds was directly proportional to the increase in incubation time (Figures 8(a)–8(f)). However, even on increasing the incubation time, more cells adhered to and spread on the nHA/PLGA scaffold compared to sHA/PLGA and PLGA scaffolds [28].

**Figure 9:**
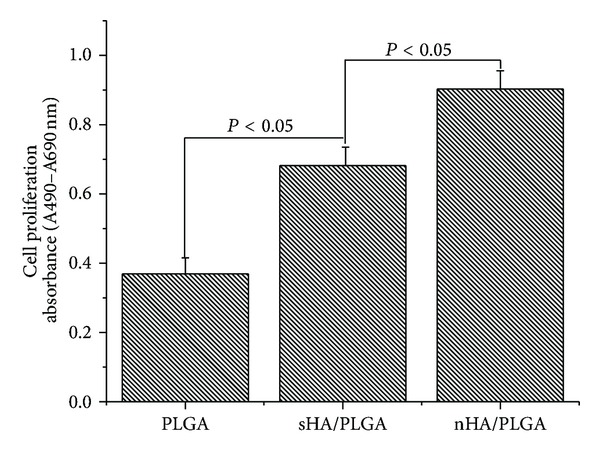
BrdU assay for the proliferation of osteoblasts on the PLGA, sHA/PLGA, and nHA/PLGA nanofiber scaffolds.

Comparable results for the difference in proliferation behavior expressed in terms of the number of newly grown cells, as determined by a BrdU assay, can be observed in Figure 9. The MC3T3 osteoblastic cells cultured on the PLGA, sHA/PLGA, and nHA/PLGA scaffolds for 48 h revealed that osteoblast cells can proliferate on all the scaffolds. However, significantly increased cell proliferation was observed on the nHA/PLGA scaffold compared to the sHA/PLGA and PLGA nanofiber scaffolds. The order of cell proliferation was nHA/PLGA > sHA/PLGA > PLGA. The enhanced cell proliferation on the nHA/PLGA scaffold (*P* > 0.05) suggests that the morphology of HA is important when applied in tissue engineering.

**Figure 10:**
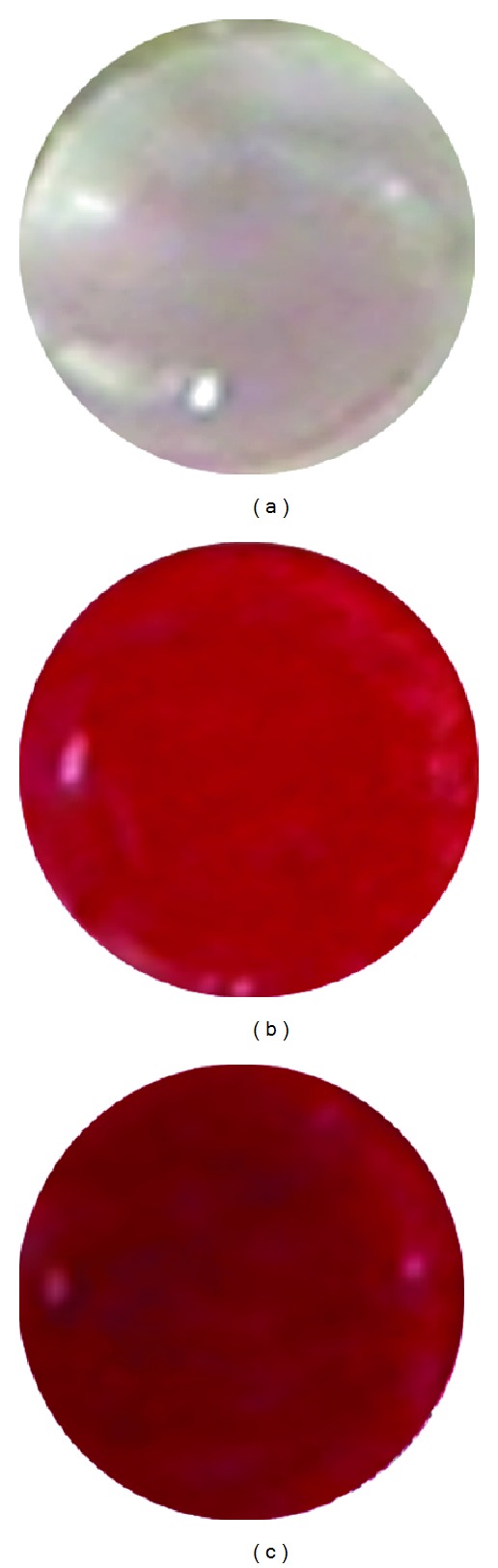
Alizarin staining of osteoblast cells cultured for 20 days on (a) PLGA, (b) sHA/PLGA, and (c) nHA/PLGA nanofiber scaffolds.

The differentiation of osteoblastic cells is one of the key factors regarding bone regeneration. Alizarin red staining is considered to be an important tool for determining the differentiation of MC3T3 osteoblastic cells (osteogenesis).  Figure 10 illustrates the osteoinductive and osteoconductive properties of MC3T3 osteoblastic cells cultured on the nHA/PLGA, sHA/PLGA, and PLGA nanofiber scaffolds for 20 days. From Figure 10 it is evident that the MC3T3 osteoblastic cells underwent osteogenesis process (i.e., laying down new bone material by MC3T3 osteoblastic cells). The osteogenesis process was determined from the appearance of a red color, which is an indicator of calcium production by MC3T3 osteoblastic cells [29]. Considering the results in Figure 10, it was cogent that nHA had a positive influence on the osteoinductive and osteoconductive properties of the MC3T3 osteoblastic cells cultured on the nHA/PLGA nanofiber scaffolds (dark red color, Figure 10(c)) when compared with the sHA/PLGA (light red color, Figure 10(b)) and PLGA nanofibers scaffolds (slightly pinkish red color, Figure 10(a)). These results have further endorsed our arguments in favor of the better bioactive properties of the nHA/PLGA compared to the sHA/PLGA and the PLGA supporting the alizarin red staining.

**Figure 11:**
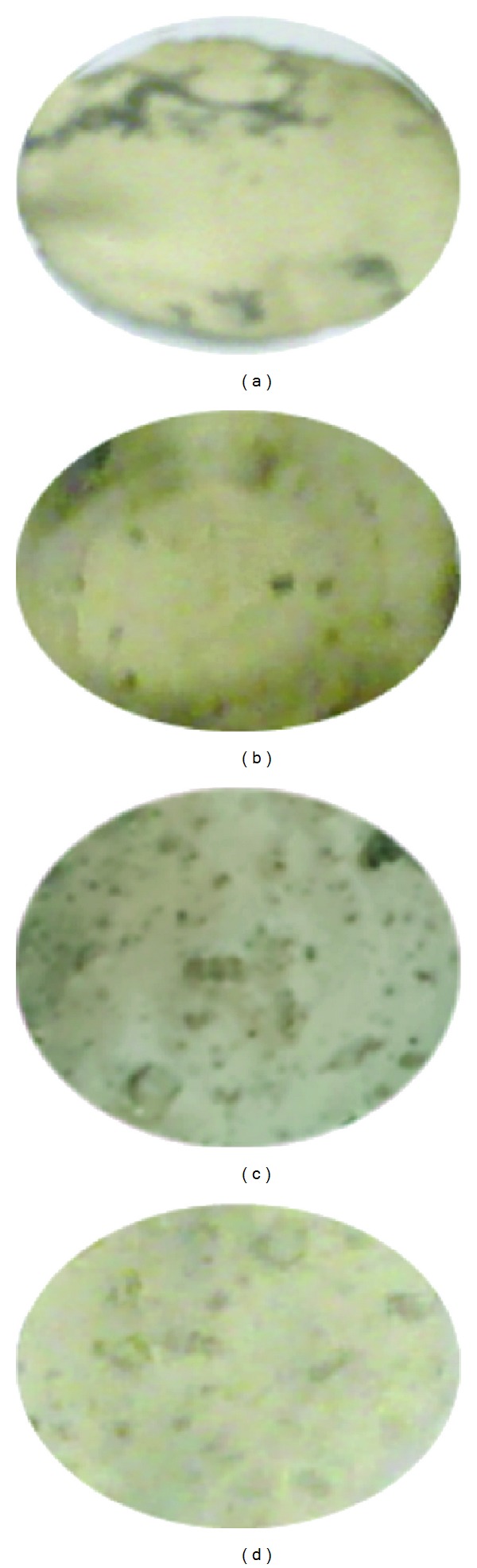
Von Kossa staining of the MC3T3 osteoblasts cultured on (a) nHA/PLGA and (b) sHA/PLGA nanofiber scaffolds cultured for 15 days and (c) nHA/PLGA and (d) sHA/PLGA cultured for 20 days.

**Figure 12:**
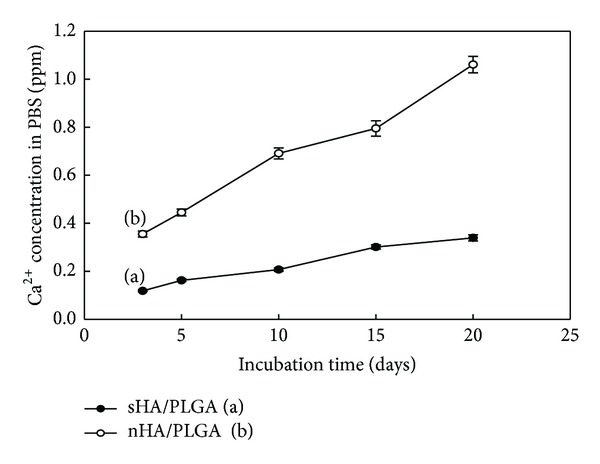
Calcium release from (a) nHA/PLGA and (b) sHA/PLGA nanofiber scaffolds as a function of incubation time.

Formation of bone nodule is considered to be the marker specific to bone differentiation. In the final stage of the cellular study assessment of the potential of nHA/PLGA and sHA/PLGA nanofiber scaffolds for the formation of bone nodule was carried out via Von Kossa assay.  Figure 11 shows the results of Von Kossa staining after culturing MC3T3 osteoblastic cells for 15 and 20 days on the nHA/PLGA and sHA/PLGA nanofiber scaffolds [8]. In Figure 11, the depicted results obtained from Von Kossa study were important in two ways. Firstly, the Von Kossa assay revealed enhanced staining on the nHA/PLGA and sHA/PLGA nanofiber scaffolds due to osteoinductive and osteoconductive properties of the HA. In Figure 11 it can be clearly observed that the calcium-containing area is stained as a silver black spot due to the replacement of calcium ions by silver ions in the presence of ultraviolet light. The increased silver black spots in the nHA/PLGA might be attributed to the better Ca^+2^ ions release of nHA in the nHA/PLGA nanofiber scaffolds (Figure 12). The increased Ca^+2^ ions production provides more ideal environments for the adhesion and proliferation of MC3T3 osteoblastic cells because calcium is the major component of bone. The increase in the number of black spots was directly proportional to the increase in incubation time. However, the results from Von Kossa assay reveal that more cells were differentiated on the nHA/PLGA (Figures 11(a) and 11(c)) nanofiber scaffold as compared to the sHA/PLGA scaffold (Figures 11(b) and 11(d)) due to much better osteoinductive and osteoconductive properties of the nHA contrary to the sHA.

Figure 12 shows the release of Ca^+2^ ions in PBS from nHA and sHA of the nHA/PLGA and sHA/PLGA nanofiber scaffolds as a function of the incubation time [22]. The release of Ca^+2^ ions from the nHA/PLGA nanofiber scaffold incubated for 20 days was faster and reached to 1.1 ppm (Figure 12(a)), whereas the amount of Ca^+2^ ions released by the sHA/PLGA nanofiber scaffold incubated for 20 days was slow and reached merely to 0.35 ppm (Figure 12(b)). The results suggest that the nHA ionizes faster compared to the sHA. The faster ionization of nHA induced a more suitable environment for the differentiation of osteoblasts because an increase in the levels of extracellular Ca^+2^ ions can increase the level of intracellular Ca^+2^ ions through the L-type and non-L-type Ca^+2^ ions channels and Ca^+2^ ions-sensing receptors. The increased level of extracellular Ca^+2^ ions induced both chemotaxis and proliferation of MC3T3 osteoblast cells [22]. They were induced by calcium-sensing receptors. The release of Ca^+2^ ions from the nHA/PLGA and sHA/PLGA composite nanofiber scaffolds can occur in the culture medium as well as in PBS. Therefore, elevated Ca^+2^ concentrations in the medium enhanced the adhesion and proliferation of cells and offered suitable conditions for cytoskeletal organization.

#### 3.5.1. Mechanism of Interaction of Charges with the Osteoblast Cell Membrane

Hydroxyapatite is vastly used as a drug delivery carrier, and the morphology of the HA has an important role to play regarding the interaction with osteoblastic cells. As HA carries different charges along *α* and *c* planes in a unit cell, that is, positive and negative, respectively, because of difference in charge there is assumption that the *α* plane tends to absorb more acidic protein, compared to that of *c* planes which tend to attract the basic ones [30, 31]. Growth of nHA along *c*-axis would thus lead to a shift toward more positively charged particles with a higher specificity of adsorption onto negatively charged acidic protein [30–32]. As biological entities are predominantly dispersed on negatively charged side, it comes as no surprise that positively charged nHA promoted good adhesion and growth contrary to its negatively charged sHA counterpart [31]. These insights can be best explained by means of Bronsted isotherm and are not necessary to have agreement with the electrophoretic studies because the rate of dissociation of HA in solution is very high, thus making it impossible to detect the charge on the HA having two different morphologies [32]. The above mentioned results obtained from the cell compatibility studies are in agreement with this concept where the nHA/PLGA composite nanofiber scaffolds offered more bioactivity and biocompatibility compared to the sHA/PLGA.

## 4. Conclusion

The nanofiber scaffolds of nHA/PLGA, sHA/PLGA, and PLGA were successfully fabricated using electrospinning technique. The nanofiber scaffolds were extensively characterized by FE-SEM, EDX, TEM, XRD, FTIR, and XPS. The potential of these scaffolds as bone regenerating material was evaluated by examining the effect of the morphology of sHA and nHA on the adhesion, proliferation, and differentiation of MC3T3 osteoblast cells. From the result it was concluded that the nHA/PLGA nanofiber scaffold showed a higher cell adhesion, proliferation, enhanced osteogenesis, and increased Ca^+2^ ions release, as detected by Brdu, alizarin red staining and Von Kossa assay. These results suggest that the nHA/PLGA nanofiber scaffold has a high potential for use in the field of bone regeneration and tissue engineering.

## Figures and Tables

**Table 1:** Atomic percent of the nHA, PLGA, nHA/PLGA, and sHA/PLGA nanofiber scaffolds calculated from survey scan spectra.

Substrates	Atomic (%)			
	C	O	Ca	P
HA	7.7	66.6	17.8	12.6
PLGA	64.61	35.39		
sHA/PLGA	59.6	35.5	3.2	4.7
nHA/PLGA	61.2	35.3	3.0	4.9
